# A simple size-tailored algorithm for the removal of chest drain following minimally invasive lobectomy: a prospective randomized study

**DOI:** 10.1007/s00464-021-08905-0

**Published:** 2021-11-30

**Authors:** Davor Stamenovic, Michael Dusmet, Thomas Schneider, Eric Roessner, Antje Messerschmidt

**Affiliations:** 1 Vidia Kliniken Karlsruhe, Karlsruhe, Germany; 2 University Thoracic Center Mainz, Mainz, Germany; 3 Royal Brompton Hospital, London, UK; 4 GFO Kliniken, Bonn, Germany

**Keywords:** Chest drain, VATS lobectomy, Minimally invasive surgery, Anatomical lung resection, Daily output

## Abstract

**Background:**

The pleural space can resorb 0.11–0.36 ml/kg of body weight/hour (h) per hemithorax. There are only a limited number of studies on thresholds for chest drain removal (CDR) and all are based on arbitrary amounts, for example, 300 ml/day. We studied an individualized size-based threshold for CDR–specifically 5 ml/kg, a simple, easily applicable measure.

**Methods:**

This is a single-center prospective randomized trial enrolling 80 patients undergoing VATS lobectomy. There were two groups: an experimental (E) group, in which once the daily output went down to 5 ml/kg the chest drain was removed and a control (C) group, with chest drain removal as per our current practice of less than 250 ml/day.

**Results:**

The groups did not differ in pre- and peri- and postoperative characteristics, except for chest drain duration (mean, SD 2.02 ± 0.97 vs. 3.25 ± 1.39 days, *p* < 0.001) and length of hospital stay (median, IQR 4.5; 3 vs. 6; 2.75 days, *p* = 0.008) in favor of E group. The re-intervention rate was the same in both groups (once in each group).

**Conclusion:**

The new threshold for chest drain removal following thoracoscopic lobectomy of 5 ml/kg/d leads to both shorter chest drainage and hospital stay without apparent increase in morbidity. (Clinical registration number: DRKS00014252).

The pleural space normally contains 0.13 ± 0.60 ml/kg of hypo-oncotic fluid (mixture of microvascular filtrates and protein at 1 g/dl) [1, 2]. The pleural fluid is produced by the parietal pleura, mainly in the less dependent parts, while absorption is by its lymphatic vessels, as well as the lymphatics of the mediastinum and diaphragm, mainly in the most dependent parts of the pleural space, maintaining an equilibrium at 0.01–0.02 ml/kg/h [3]. However, if a pathological process is present (tumor involvement, inflammation, postoperative status after thoracic surgery), the effusion may come from visceral pleura, diaphragm, and peritoneum as well [4]. A tenfold increase in pleural fluid production only leads to a 15–20% increase in the volume of the pleural effusion [5].

Applying this to a patient weighing 70 kg, approximately 470 ml of effusion will be produced and absorbed daily in the pleural space or approximately 0.11–0.36 ml/kg of body weight/h per hemithorax [6, 7].

At present most decisions regarding CDR are arbitrary and vary widely between institutions both nationally and internationally. In Germany, these typically range between 100 and 300 ml/d [8], while in Denmark this seems to differ with an accepted threshold of up to 400 ml/d [9, 10].

There are a limited number of publications available on the criteria for chest drain removal (CDR) after video-assisted thoracoscopic (VATS) lobectomy based on the output through the chest drain (CD), only one of which was a prospective randomized controlled study [11]. However, there are currently no prospective randomized studies of the fluid output per 24 h for CDR removal after VATS lobectomy based on the patient’s size.

## Objectives

The primary objective is to examine a feasibility and safety of an algorithm with CDR once the daily output goes down to 5 ml/kg/day.

There were two primary endpoints of this study: the chest drainage duration (CDD) and the number of re-interventions caused by inadequate drainage of the pleural space.

The incidence of re-interventions (RI) will be used to calculate the number of the patients needed to be enrolled in a non-inferiority study aiming to reach 0.8 power for the maximum of 10% higher re-interventions rate in the experimental group compared with the active control group (250 ml/d).

The secondary endpoint is the rate of complications in both groups.

## Materials and methods

This is a single-center prospective randomized pilot trial registered at the Germany register for clinical studies (DRKS00014252) and approved by the ethical committee of University of Heidelberg (S-159/2018). The study was conducted in accordance with the Declaration of Helsinki, and written informed consent was obtained from all patients.

Based on the historical data from our institution on successive 148 patients, who have undergone VATS lobectomy and a standard deviation derived from the CDD (SD = 1.475), 72 patients (36 in each group) are required for the study to achieve a power of 0.8 specifically for the one-day difference in CDD between the groups.

### Randomization

The numbers 1 to 100 are placed in an arbitrary order using an Excel random number generator. An independent, non-medical person transfers these numbers onto a sheet of paper. These sheets are then placed in envelopes numbered 1 to 100 and sealed. (Example: envelope number 5 contains number 40).

Once the eligibility criteria have been met, an envelope corresponding to the particular patient (the fifth envelope belongs to the 5th enrolled patient, 10th to the 10th, etc.) is unsealed and a number within revealed. Should an even number appear, that patient belonged to the experimental group. Patients with odd numbers went into the control group.

Eighty patients undergoing VATS lobectomy were randomized to the two groups: the experimental (E) group, with CDR once the output dropped to 5 ml/kg/d and a control (C) group, with CDR at less than 250 ml/d.

All patients over 18 years of age, capable of giving consent and undergoing a minimally invasive lobectomy (for benign disease, primary lung cancer, or a pulmonary metastasis from another primary tumor), were eligible for the study. The diagnosis of the lesion could be obtained pre or intraoperatively.

Exclusion criteria were any other type of resection or utilization of an alternative approach (including conversion thoracotomies), pleural carcinosis, any air leak, or revision surgery within the first 24 h postoperatively (for example for bleeding), heart or kidney failure, as well as BMI > 30 kg/m^2^ or < 18 kg/m^2^.

### Operative management

VATS lobectomy with systematic lymph node dissection was performed by means of three, two, or single port incisions at the surgeon’s discretion. A single chest drain (24 F, Rocket Medical, Washington, DC, USA) was placed in the pleural space at the end of the procedure in every case.

On the 1st postoperative day, clinical data were recorded followed by randomization of the patient into the allocated treatment group provided there was not an on-going air leak.

The postoperative care of the patients was standardized with at least daily visits starting on postoperative day (POD) 1 in the intermediate care unit (IMC), as well as on the ward until discharge. Fluid output was recorded daily by the nursing staff at 7 am. Chest X-rays (PA and lateral) were performed the day after CDR and were reviewed by two physicians (one of which was the patient’s surgeon). If the patient was well and the X-rays showed no complications the patient was in principle discharged home.

The patients were monitored daily while in hospital and for 2 weeks following discharge. All complications were recorded, specifically the need for CD reinsertion or for thoracentesis or the development of pleural empyema or any need of antibiotic treatment for postoperative pneumonia. Complications were defined according to the definitions from the Society of Thoracic Surgeons and The European Society of Thoracic Surgeons [12].

Patients were instructed to seek for help from our department, either directly at the outpatient clinic, emergency room, or by calling, in particular if they had any respiratory problems.

The patients were reviewed in the outpatient clinic at approximately two weeks after discharge. In addition to clinical (e.g., new dyspnea) and/or radiological findings, an ultrasound examination was routinely carried out. Re-intervention (thoracentesis, CD reinsertion, or thoracoscopy) was carried out if there was a pleural effusion of more than 500 ml and if the patient complained of dyspnea: The risks of the evacuation of a pleural effusion of less than 500 ml usually outweigh the benefits [13] and these small effusions are sometimes difficult to visualize with an X-ray [14].

The amount of effusion was determined by means of an ultrasound examination: The sum of the basal distance between the lungs, the diaphragm, and the lateral height of the effusion multiplied by 70 gives a good estimate of the volume of the effusion (*r* = 0.87) [15, 16].

### Statistical analysis

All but one of the continuous variables were normally distributed and therefore compared by means of Student’s t test. The single continuous non-normally distributed variable was the length of hospital stay and Wilcoxon’s test was utilized for comparison. Categorical variables were analyzed using Fisher’s test in case of two variables and Wald test if more than two needed to be analyzed.

Data were analyzed using R stats [17], Microsoft Excel®, and G-Power [18].

## Results

**Fig. 1:**
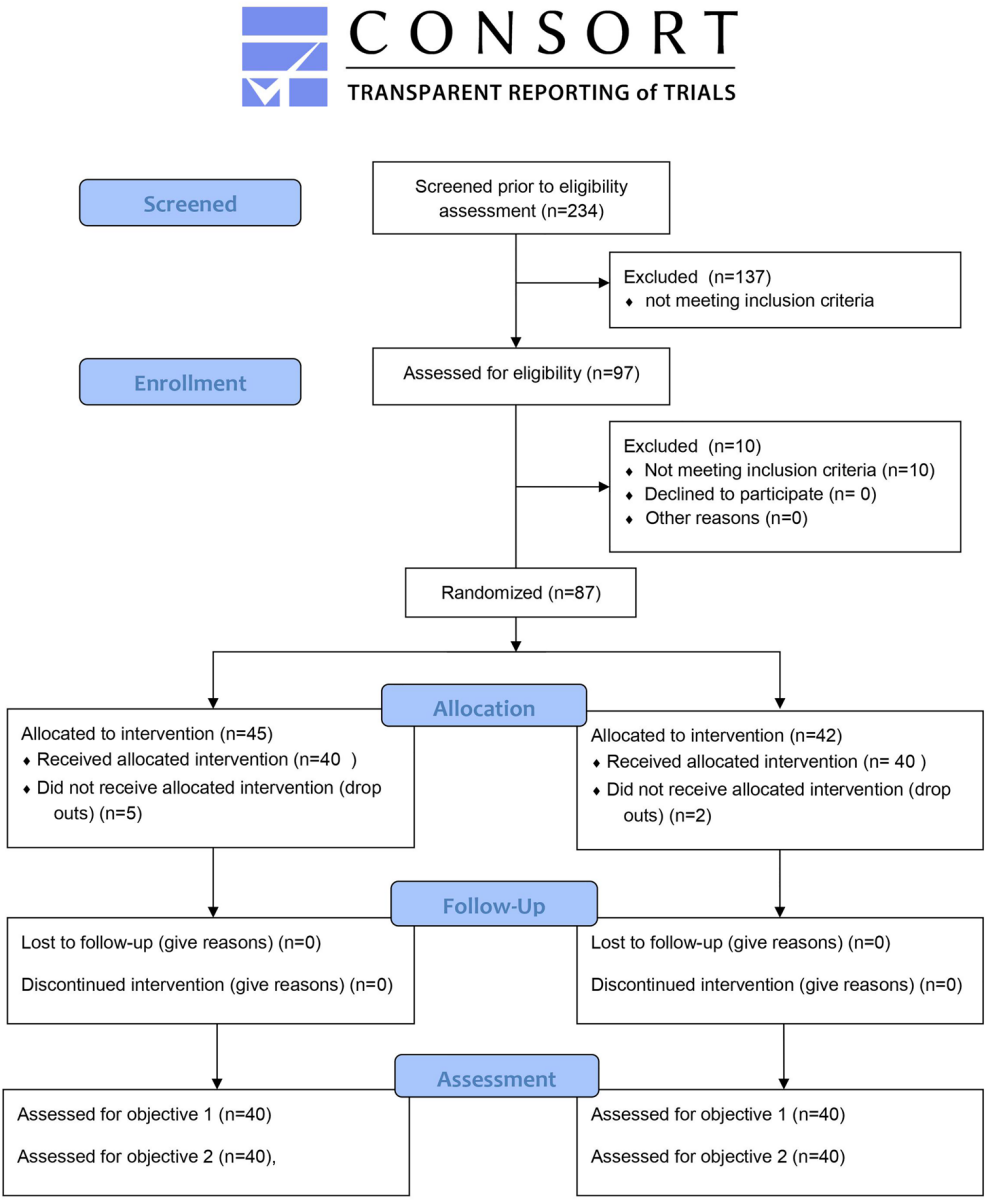
CONSORT flow diagram

From April 2018 to December 2019, 87 patients underwent randomization after fulfilling the entry criteria. Seven patients were excluded after randomization (Fig. 1) [19]. All of them were men: one patient developed an air leak on the 2nd postoperative day, four were diagnosed with heart failure (BNP > 15.000), one was excluded due to a breach of the study protocol (violation of the time of CDR), and one developed pneumonia, bronchopleural fistula (BPF), and pleural empyema.

The two groups were very similar, as shown (Table 1).

**Table 1:** Demographic characteristics of cohorts

Characteristic	Group E (*n* = 40)	Group C (*n* = 40)	*p*
Age (mean, SD)	66 (9)	68 (12)	0.53
Sex (male, percentage)	28 (70%)	25 (62.5%)	0.64
FEV1 (mean, SD)	0.82 (0.24)	0.84 (0.18)	0.67
DLCO (mean, SD)	0.7 (0.23)	0.73 (0.19)	0.54
ASA score (median, range)	3 (2–4)	3 (2–4)	0.46
BMI (mean, SD)	25.2	24	0.25
Preoperative CT (%)	5 (12.5%)	6 (15%)	1
Actual smoker (%)	11 (27.5)	15 (37.5%)	0.47

*E* experimental; *C* control; *SD* standard deviation; *FEV*_1_ forced expiratory volume in 1 s; *DLCO* diffusing capacity; *ASA* American Society of Anesthesiologists; *BMI* body mass index; *CT* chemotherapy

**Fig. 2:**
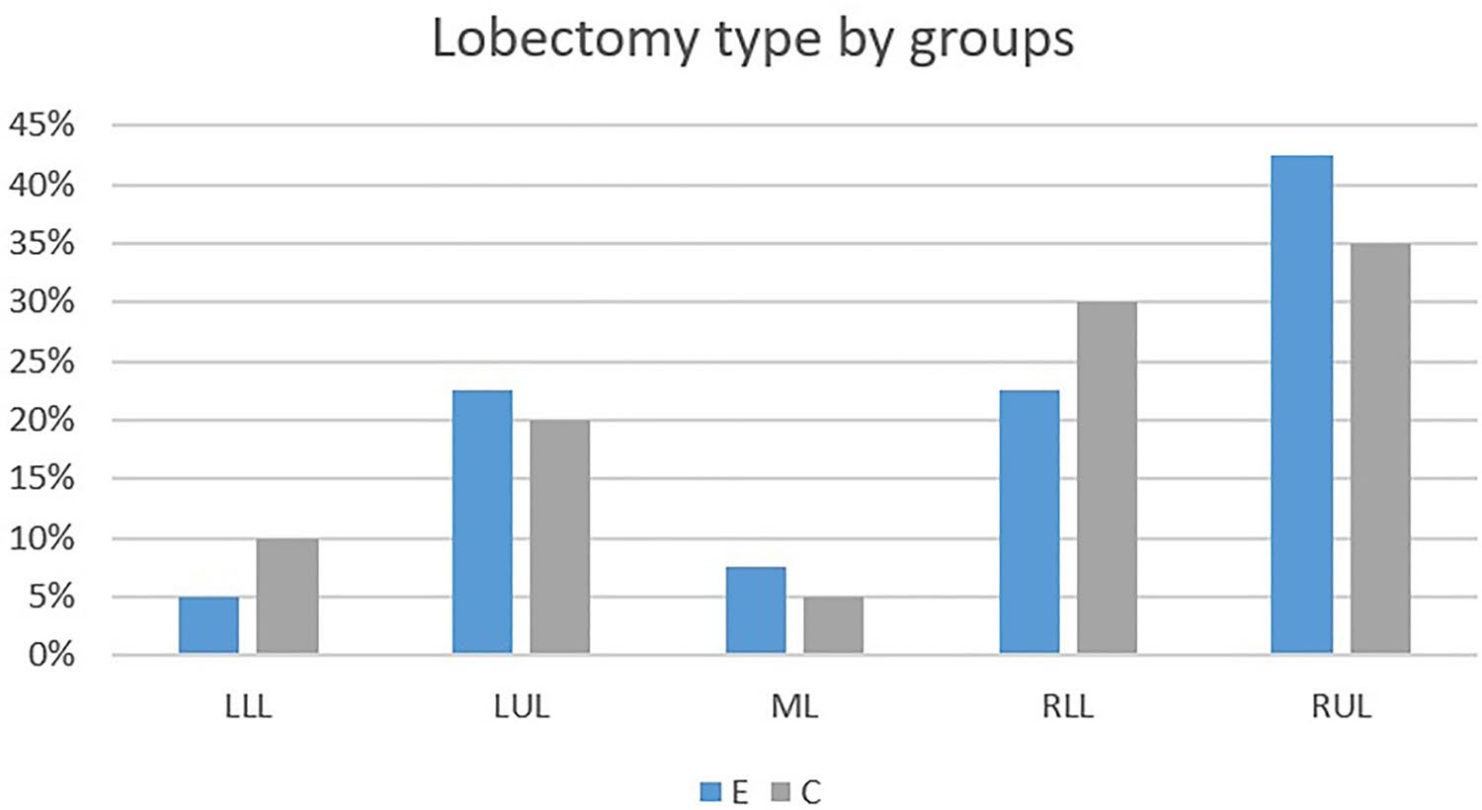
Lobectomy by groups. *LLL* left lower lobectomy; *LUL* left upper lobectomy; *ML* middle lobe lobectomy; *RLL* right lower lobectomy; *RUL* right upper lobectomy; *C* control group; *E* experimental group

The perioperative characteristics of the two patient groups were very similar (Table 2; Fig. 2). We also looked at how much adhesiolysis was required. This varied from none, around one lobe, and around more than one lobe and this was not significantly different between the two groups (*p* = 0.75). We also looked at the underlying disease process (benign disease vs primary lung cancer vs metastatic disease) and again there were no significant differences between the two groups (*p* = 0.81).

**Table 2:** Perioperative characteristics of cohorts

Characteristic	Group E (*n* = 40)	Group C (*n* = 40)	*p*
OP length, min (mean, SD)	162 (35)	155 (38)	0.39
pT, cm (SD)	3.1 (1.4)	2.9 (1.5)	0.47
Nr. LN removed (SD)	18 (7)	21 (8)	0.2
Complications (%)	7 (17.5)	9 (22.5)	0.32
Chest drain duration, days (mean, SD)	2.02 (0.97)	3.25 (1.39)	0.000
LOS, days (median, IQR)	4.5 (3)	6 (2.75)	0.008
Nr. of re-intervention (%)	1 (2.5)	1 (2.5)	1

*E* experimental; *AC* active control; *OP* operation; *pT* pathological tumor size; *SD* standard deviation; *Nr*. number; *LN* lymphatic nodes; *LOS* length of hospital stay; *IQR* interquartile range

The difference in CDD of 1.22 days (29.4 h) in favor of the experimental treatment is highly significant (*δ* = 1.02011). Both the upper limit of the 95% CI (− 0.6894) which is lower than 0 and the results of t test (t (69.81) = − 4.562, *p* = 0.001 show superiority of the experimental treatment for CDD compared to the control treatment. The power of this results, as the probability of rejecting a false Ho is very high at 0.99.

There was one re-intervention in each group: in the C group, an 86-year-old female patient underwent a reinsertion of the CD on the eighth postoperative day (POD)—the fourth day after CDR—due to a parapneumonic effusion. In the E group an 81-year-old female patient was re-drained on the second postoperative day—a day after CDR occurred for symptomatic pleural effusion.

However as this study was not powered to study the rate of re-intervention (this would have required 280 patients) this result is not conclusive.

Apart from these two re-interventions, there was another case with pleural effusion, belonging to the C group, at the routine postoperative control at outpatient clinic two weeks after the patient was discharged. Despite the amount of pleural effusion (500 ml), the patient was asymptomatic and thus no intervention was performed.

The complication rate was comparable and nonsignificantly higher in the C group with a higher incidence of pneumonia than in the E group (4 vs. 2) as shown in Table 3. There were more cases of atrial fibrillation in the E group (3 vs 1). The calculated relative risk of having complications in the control group was 1.286 compared to the E group.

**Table 3:** Complications rate in both groups

Diagnosis	E Group (7/40)	C Group (9/40)
Exacerbation COPD	1	1
Atrial fibrillation	2	1
Pneumonia	2	4
Delirium	1	1
Pneumonia and atrial fibrillation	1	0
Respiratory failure	0	1
Acute kidney failure	0	1

*COPD* chronic obstructive pulmonary disease; *E* experimental; *C* control

As mentioned, one patient (group C) developed a BPF and was excluded from the analysis. There were no deaths in either study group (either in hospital or during the 2 weeks of follow-up).

Finally, four patients were readmitted within first two weeks after discharge from hospital: three in group C and one in group E.

Control group: one patient was readmitted on day 4 post-discharge to drain a pneumothorax (no effusion present). Another patient was readmitted 2 days post-discharge with respiratory failure (exacerbation of COPD). The 3rd patient was readmitted day 7 post-discharge for treatment of a pulmonary embolism.

Experimental group: one patient was readmitted 3 days post-discharge for pain management and a wound infection.

## Discussion

This study excluded patients with any air leak beyond 24 h after surgery, allowing us to focus on the sole criteria of chest drain output as a criterion for chest drain removal.

Chest drains are routinely placed into the pleural space following lobectomy by the vast majority of thoracic surgeons for safety (to prevent tension pneumothorax) and to have insight into possible complications, such as postoperative bleeding.

Chest drain removal favors better mobilization, reduced pain, and thereby shorter length of hospital stay—which all may potentially promote both lower complication rates and hospital costs, However there is poor consensus about the criteria for CDR when there is not an air leak.

The one prospective randomized trial used absolute thresholds of 150, 300, and 450 ml/d for CDR and found that 300 ml/d was the safe threshold [11].

Other randomized controlled studies (RCT) to answer different questions related to CDR after lung surgery used different thresholds for CDR. In one study [20] this threshold was established as 250 ml, in another [21] it was as 200 ml. A third study [9] puts up no upper limit prior to CDR, demonstrating, however, in nearly 10% of patients a need for immediate re-intervention, either by means of CD reinsertion or thoracentesis. In none of these studies was there any information about the patients after discharge (specifically if subsequent pleural intervention was required).

The aim of this RCT was to explore a threshold for CDR based on the patient’s size or more specifically weight as a surrogate for size.

Obesity is a potential confounder because obese patients actually have a smaller pleural space than slim patients (the diaphragm is pushed up by the obese abdomen), but their weight alone would indicate a larger pleural space. The BMI would therefore theoretically be a better index than the patient’s weight. However, height and weight are readily available, whereas in most hospital systems the BMI is either harder to find or needs to be calculated. In addition, 5 ml/kg is very easy to calculate and can be done by most people without a calculator. Our priority was to have a simple, easy method that is highly reproducible. The average BMI of our patients was close to the German average for patients of this age [22] and is already on the cusp of being overweight (BMI between 25 and 30) with an average BMI of 25.2 (group E) and 24 (group C). In obese patients (BMI > 30), 20 kg can perhaps be taken off of their body weight prior to making this calculation if this is a concern [23].

The intra-pleural re-intervention rate was identical in both groups at one each. The power calculations for this study were based on CDD and it is entirely possible that this study was underpowered to address this issue in a robust manner.

We included over 10% more patients than our power calculations mandated (80 vs 72), so the conclusion of our study that weight-based CDR at 5 ml/kg/d is beneficial in terms of CDD is both statistically and clinically highly significant as well as valid.

We also have performed systematic lymph node dissection at all of the enrolled patients, only one appearing to have a benign disease afterward (lung abscess).

The LOS was 1.5 days shorter in the experimental group and this was a statistically significant finding. However, the study was not powered for this criteria (we would have needed 280 cases for this). Moreover, as part of the protocol the patients should have been discharged on the day following CDR, which had not occurred in all patients as per protocol. There are two principle reasons for these delays: in Germany the hospital loses money if the patient stays less than 4 days and some patients feel they need an extra day to feel ready to go home. If either of these events occurred, we did not exclude the patients from the study. Thus the LOS findings are less robust than the CDD findings. Nonetheless, in practice and in most publications CDR and LOS are usually highly correlated [24–26].

In conclusion, this study shows that CDR as soon as drainage is less than 5 ml/kg of body weight per day leads to reduction in CDD as well as in LOS. Further study may be necessary to confirm a non-inferiority of the new threshold regarding the need for intra-pleural re-intervention.
